# Combination of dual JAK/HDAC inhibitor with regorafenib synergistically reduces tumor growth, metastasis, and regorafenib-induced toxicity in colorectal cancer

**DOI:** 10.1186/s13046-024-03106-8

**Published:** 2024-07-11

**Authors:** Prachi Bajpai, Sumit Agarwal, Farrukh Afaq, Sameer Al Diffalha, Darshan S. Chandrashekar, Hyung-Gyoon Kim, Abigail Shelton, C. Ryan Miller, Santosh K. Singh, Rajesh Singh, Sooryanarayana Varambally, Ganji Purnachandra Nagaraju, Ashish Manne, Ravi Paluri, Moh’d Khushman, Upender Manne

**Affiliations:** 1 Department of Pathology, University of Alabama at Birmingham, Birmingham, AL USA; 2 O’Neal Comprehensive Cancer Center, University of Alabama at Birmingham, Birmingham, AL USA; 3 Department of Microbiology, Biochemistry & Immunology, Morehouse School of Medicine, Atlanta, GA USA; 4 Department of Medicine, University of Alabama at Birmingham, Birmingham, AL USA; 5 Department of Internal Medicine, The Ohio State University Comprehensive Cancer Center, Columbus, OH USA; 6 Department of Hematology and Oncology, Wake Forest School of Medicine, Winston-Salem, NC USA; 7 Department of Medicine, Washington University in St. Louis/Siteman Cancer Center, St. Louis, MO USA

**Keywords:** Colorectal cancer, JAK/HDACi, Regorafenib, Combination therapy, Toxicity

## Abstract

**Background:**

Treatment with regorafenib, a multiple-kinase inhibitor, to manage metastatic colorectal cancers (mCRCs) shows a modest improvement in overall survival but is associated with severe toxicities. Thus, to reduce regorafenib-induced toxicity, we used regorafenib at low concentration along with a dual JAK/HDAC small-molecule inhibitor (JAK/HDACi) to leverage the advantages of both JAK and HDAC inhibition to enhance antitumor activity. The therapeutic efficacy and safety of the combination treatment was evaluated with CRC models.

**Methods:**

The cytotoxicity of JAK/HDACi, regorafenib, and their combination were tested with normal colonic and CRC cells exhibiting various genetic backgrounds. Kinomic, ATAC-seq, RNA-seq, cell cycle, and apoptosis analyses were performed to evaluate the cellular functions/molecular alterations affected by the combination. Efficacy of the combination was assessed using patient-derived xenograft (PDX) and experimental metastasis models of CRC. To evaluate the interplay between tumor, its microenvironment, and modulation of immune response, MC38 syngeneic mice were utilized.

**Results:**

The combination therapy decreased cell viability; phosphorylation of JAKs, STAT3, EGFR, and other key kinases; and inhibited deacetylation of histone H3K9, H4K8, and alpha tubulin proteins. It induced cell cycle arrest at G0-G1 phase and apoptosis of CRC cells. Whole transcriptomic analysis showed that combination treatment modulated molecules involved in apoptosis, extracellular matrix-receptor interaction, and focal adhesion pathways. It synergistically reduces PDX tumor growth and experimental metastasis, and, in a syngeneic mouse model, the treatment enhances the antitumor immune response as evidenced by higher infiltration of CD45 and cytotoxic cells. Pharmacokinetic studies showed that combination increased the bioavailability of regorafenib.

**Conclusions:**

The combination treatment was more effective than with regorafenib or JAK/HDACi alone, and had minimal toxicity. A clinical trial to evaluate this combination for treatment of mCRCs is warranted.

**Supplementary Information:**

The online version contains supplementary material available at 10.1186/s13046-024-03106-8.

## Background

In the United States, colorectal cancer (CRC) is the second leading cause of cancer mortality; in 2023, an estimated 153,020 new cases and 52,550 deaths, were predicted [1]. The prognosis of metastatic CRC (mCRC) is poor. Despite all the advances in the treatment of CRC, the 5-years survival to date is still around 14% [1, 2]. Although treatment of early stages of CRC typically includes surgical resection, mCRCs are treated mainly with systemic therapy including, but not limited to, chemotherapy, targeted therapy and immune check point inhibitors [2]. In the last two decades, median overall survival (OS) for mCRC patients has increased from less than 1 year to around 30 months due to advancements in early and late line treatment options [3–7]. For CRC, early lines chemotherapy regimens include fluorouracil (FU)-based regimens, which include FOLFOX (folinic acid, FU, and oxaliplatin) and FOLFIRI (folinic acid, FU, and irinotecan), with or without targeted biologic agents such as cetuximab and panitumumab, which are anti-EGFR agents or bevacizumab (a VEGF inhibitor) [8, 9]. In 2012, the US FDA approved regorafenib, which targets several receptor tyrosine kinases (RTKs), including fibroblast growth factor receptors (FGFRs), platelet-derived growth factor receptor beta (PDGFRβ), epidermal growth factor receptor (EGFR), and extracellular signal-regulated kinase (ERK1/2) [10], for treatment of mCRC patients who fail FU-based chemotherapy regimens [11, 12].

Several clinical trials have evaluated regorafenib in previously treated mCRC patients. CORRECT and CONCUR phase III clinical trials have shown modest OS benefit compared to placebo. The most common grade 3 and 4 adverse events were hand-foot skin reaction, fatigue diarrhea, hypertension, and rash and that led to treatment delay, dose reduction or discontinuation [13, 14]. CONSIGN and REGARD single arm clinical trials results were consistent with the known safety profile of regorafenib [15, 16]. Though regorafenib has anti-angiogenic and anti-proliferative properties, it also exhibits high toxicity and poor tolerability for patients [17], effects that were evident in the first cycle of treatment [18]. Further, to optimize regorafenib treatment and to increase tolerability, a randomized phase II, ReDOS trial was conducted in previously treated mCRC. Fewer adverse effects were evident in the dose-escalation group [19, 20]. JAK and HDAC inhibitors have been explored individually as potential therapeutic agents for various solid tumors with limited efficacy [21, 22]. A bifunctional/hybrid small-molecule Janus kinase/Histone deacetylase inhibitor (JAK/HDACi) demonstrates co-inhibition of HDAC and JAK kinases, resulting in suppression of progression of hematologic and solid tumors [23, 24]. JAK/HDACi, a potent JAK1-3/HDAC2/6 dual inhibitor, exhibits antiproliferative and proapoptotic activities [23, 25]. Thus, we initiated a preclinical investigation to evaluate the effect of regorafenib in combination with JAK/HDACi and to assess whether this combination reduces regorafenib-induced toxicity in CRC and enhances its therapeutic efficacy in combination with JAK/HDACi at lower drug concentrations. Advantages/rationales to use this hybrid molecule are: a) concurrent inhibition of enzymes of JAK and HDAC families, which are activated in CRC [26–30], by JAK/HDACi could enhance the anti-tumor immunity, modulate expression profiles of genes involved in cell cycle regulation and apoptosis, and result in synergistic effects, leading to enhanced efficacy of regorafenib; b) the combination of regorafenib with JAK/HDACi may lead to changes in the epigenetic landscape of CRC, potentially reactivating silenced tumor suppressor genes and may inhibit additional kinases; and c) combining JAK/HDACi with regorafenib may inhibit other signaling pathways activated in CRC. In the current study, we evaluated the effects of this combination in enhancing the therapeutic efficacy and reducing regorafenib-induced toxicity, using in vitro and in vivo CRC preclinical models, which include patient-derived xenografts (PDXs), luciferase tagged HT29 experimental metastasis, and MC38 syngeneic mouse models. Our results indicate that the efficacy of regorafenib in combination with JAK/HDACi has a synergistic inhibitory effect on tumor growth, and metastasis, with no apparent toxicity.

## Methods

### CRC clinical tissue specimens

Frozen archival CRC tissues, along with their uninvolved adjacent noncancerous/tissue specimens, were procured through the Anatomic Pathology Division of the University of Alabama at Birmingham (UAB). Following guidelines described by the Declaration of Helsinki, Institutional Review Board (IRB#090513004) approval from UAB was obtained for experimental use of specimens. Lysates for tumor (T) and adjacent uninvolved noncancerous tissue (N) were used for protein analyses.

### Cell lines and culture media

Normal primary colonic epithelium cells were obtained from Cell Biologics, and CRC cell lines (HCT116, SW480, HT29, and RKO) were procured from American Type Culture Collection (ATCC). The CRC cell lines exhibiting various statuses of TP53, KRAS, BRAF, PIK3CA, PTEN and Microsatellite status: stable (MSS)/Instable (MSI) summarized in Table S1, were grown in McCoy’s medium (Fisher Scientific, Hampton, NH), and normal primary colonic epithelium cells were grown in human epithelial cell medium (Cell Biologics, Chicago, IL). The cells were STR profiled and were tested for mycoplasma contamination. Culture media were supplemented with 10% fetal bovine serum and penicillin–streptomycin, and cells were incubated at 37ºC with 5% CO_2_.

### Treatment with JAK/HDAC inhibitor and regorafenib

We tested JAK/HDACi (Cat. # HY-126141) and regorafenib (Cat. # HY-10331) obtained from MedChem Express (Monmouth Junction, NJ). Both drugs were dissolved in DMSO, and 5 mM stock was prepared for cell culture experiments. For animal experiments, drugs were prepared according to the vender’s suggestions.

### Cell viability assay

The viability of normal primary colonic epithelium cells were determined after 72 h of treatment, following procedure described in our previous study [31]. Concentration-dependent dose titrations in normal primary colonic epithelium cells with a range of concentrations from 250 nM-5 µM were conducted, with JAK/HDACi, regorafenib and combination treatment. In addition, to evaluate the efficacy of drugs in CRC cells, 3-(4,5-dimethylthiazol-2-yl)-2,5-diphenyltetrazoliumbromide** (**MTT) experiment were conducted at 500 nM concentration. Further in vitro experiments in CRC cells were conducted at 500 nM concentration.

### Colony formation assay

To assess the colony formation after drug treatment, 1,000 CRC cells in triplicate were plated into 6-well plates, after treatment JAK/HDACi, regorafenib, and their combination. After 10 days, cell colonies were fixed with 5% glutaraldehyde and stained with crystal violet (Sigma-Aldrich). Quantification of colonies was performed by the 10% acetic acid extraction method described by Kueng and coworkers [32].

### Kinome analysis

SW480 and RKO cells were treated with vehicle (DMSO), JAK/HDACi, regorafenib, and their combination for 45 min, and cell lysates were prepared. Tyrosine kinase (PTK) activity (kinomic) profiling was performed using the PamStation®12 platform (PamGene, BV, The Netherlands). As per standard kinomic protocol as described previously [33]. Whole-chip comparative analysis (BioNavigator Upkin PTK v8.0) was performed between groups generating Mean Kinase Statistics (direction of change).

### Immunoblot analysis

Western blot analyses were performed using NuPAGE 4–12% Bis–Tris Midi Protein Gels, (Thermo Fisher Scientific, Waltham, MA), following procedure described in our previous study [31]. Details of antibodies used in the study is provided in Table S2. The Super Signal West Femto Maximum sensitivity substrate (EMD Millipore, Rockford, IL) was used to detect signals. The signal was detected with an Amersham Imager 600RGB (GE Healthcare Life Sciences).

### Whole transcriptomic sequencing and Assay for Transposase-Accessible Chromatin with sequencing (ATAC-seq)

A total of 1 × 10^6^ cells/dish were seeded for RNA extraction from CRC cells after 12 h of treatment with JAK/HDACi, regorafenib, and their combination with Trizol, following the manufacturer’s instructions (Invitrogen). Poly A enrichment from total RNA was performed, and library were prepared using cDNA synthesis, cDNA library preparation kits from Agilent Technologies. Final library were run on Illumina Nextseq 550 sequencer using Illumina NextSeq 500/550 High Output Kit, as 75-bp single-end reads. For RNA sequencing data analyses approaches were followed as described in our prior studies [34, 35]. For ATAC-seq, 100,000 cells were counted after 6 h drug treatment, and treated with DNase solution following the vendor’s protocol, and submitted to Active Motif (Carlsbad, CA) for library preparation, sequencing, data processing, analyses, and visualization.

### cDNA synthsis and Quantitative PCR (qPCR)

High-capacity cDNA reverse transcription kit with RNase inhibitor (Applied Biosystems, Thermo Fisher Scientific, Waltham, MA) was used to reverse transcribed 2 µg of total RNA. Quantification of gene targets was done with10 ng cDNA, for validation of RNA-seq targets using PowerUp SYBR green master mix (Applied Biosystems, Thermo Fisher Scientific, Waltham, MA) on an ABI real-time PCR machine and analyzed using Quant-studio Real-Time PCR software (Applied Biosystems). List of primers are provided in Table S3.

### Immunohistochemical analysis

The immunophenotypic expressions of Ki67, in sections from PDX tissues, and CD8, CD45, pSTAT3^Y705^, pERK1/2 in tumor sections collected from immunocompetent mice were assessed by immunohistochemical (IHC) assays as described earlier [36].

### TUNEL staining

Apoptosis induced in a PDX model by JAK/HDACi, regorafenib and their combination treatments was analyzed by staining FFPE sections using TACS-2 TdT-DAB In Situ Apoptosis Detection Kit (R&D Biotechne, Minneapolis, MN), following the manufacturer’s instructions.

### Cell cycle analysis

To evaluate the effect of drugs on cell cycle arrest, SW480 and RKO cells were seeded, 1 × 10^6^/dish in triplicates in four groups: vehicle, JAK/HDACi, regorafenib, and their combination (for 24 h, following procedure described in our previous study [31]. Cell cycle analysis was performed using BD LSR FortessaTM Flow Cytometer (BD Biosciences, Mountain View, CA).

### Flow cytometry for quantification of apoptosis

To evaluate the effect of drugs on apoptosis, cells were stained by use of TACS Annexin V-FITC apoptosis detection kit obtained from R&D Systems (Minneapolis, MN), following the manufacturer’s protocol. CRC cells (HCT116, RKO, HT29, and SW480) were seeded at 1 × 10^5^/dish in triplicates in four groups: vehicle, JAK/HDACi, regorafenib, and the combination for 72 h. The cells were analyzed with a BD LSR FortessaTM Flow Cytometer (BD Biosciences, Mountain View, CA).

### Drug efficacy on patient-derived xenografts (PDXs)

To assess the effect of individual and combination drug treatments on tumor growth, a PDX that was developed from histologically confirmed colon cancer tissue, obtained from the Department of Surgery at UAB. It was used after approval of the UAB Institutional Animal Care and Use Committee of the University of Alabama at Birmingham (Animal Project Number (APN): IACUC-20207). The details of the PDX model development is described in our publication [36]. Animals were treated with vehicle (*n* = 4), JAK/HDACi (*n* = 4; 30 mg/kg body wt), regorafenib (*n* = 4; 6 mg/kg body wt), or the combination (*n* = 4; JAK/HDACi: 30 mg/kg body wt + regorafenib: 6 mg/kg body wt) every third day and tumor were measured weekly. Mice were sacrificed upon completion of experiment and tumors were harvested, photographed, weighed, and processed for FFPE block preparation.

### Efficacy of combination therapy in an experimental metastasis model

To assess the effect of JAK/HDACi, regorafenib and their combination drug treatments on metastasis, an experiment was performed with 6-week-old NOD/SCID/IL2γ receptor-null (NSG) mice (male and female) as described [37]. All procedures and experimental protocols were approved and conducted in compliance (IACUC-21501). For metastasis experiments, as described previously [31], tail vein injections with HT29 cells (0.5 × 10^6^) carrying a luciferase expression construct were conducted. Mice were imaged at 0, 10, and 30 days post injection, and treated with vehicle or drugs (with JAK/HDACi, regorafenib and their combination). Drug treatments were performed as described in PDX experiment. Bioluminescence imaging was accomplished using an IVIS Lumina III instrument (Perkin Elmer, Waltham, MA), and mice were injected with luciferin 10 min prior to imaging. After 30 days, mouse lungs and kidneys were dissected, and ex vivo analyses were performed.

### Syngeneic mouse model to evaluate the efficacy of combination therapy

To examine the effect of individual and combination treatments on immunomodulation, MC38 (0.25 × 10^6^) were injected into male and female immunocompetent C57BL/6 mice that were obtained from the Jackson Laboratory. Drug treatments were done in similar manner as described in PDX experiment. All procedures and experimental protocols were approved and conducted in compliance (IACUC-21501). The animals were sacrificed upon completion of the experiment, and tumor tissue was harvested and processed for, RNA, protein isolation and FFPE block preparation.

### NanoString nCounter differential gene expression analysis

Gene expression was quantified digitally using the nCounter Gene Expression Assay (NanoString Technologies, Seattle, WA) by mouse PanCancer IO 360. Assays were performed according to the manufacturer’s instructions. The hybridized samples were loaded onto the nCounter PrepStation with automatic processing. The raw counts of expression data files were analyzed using the Rosalind web platform.

### Toxicity analysis in serum samples

Upon completion of the experiment for 30 days in HT29 metastasis model, before mice were sacrificed, blood was collected and serum was isolated to evaluate liver and kidney functions to assess toxicity with JAK/HDACi and regorafenib alone, and their combination treatment, using a comprehensive Panel EWrap (Cat# 6330-COMP) by a DRY CHEM 4000 Analyzer.

### Cytokine analysis in serum samples of C57BL/6 mice

Cytokines were measured in serum samples of syngeneic mouse, treated with JAK/HDACi and regorafenib, and their combination along with vehicle control, using MesoScale Discovery (Rockville, MD) mouse V-Plex Proinflammatory Panel I kit with a SIRRUS Stanbio automated chemical analyzer.

### Pharmacokinetic (PK) profiles of regorafenib and JAK-HDACi

Blood samples were collected from C57BL/6 mice treated with regorafenib and JAK-HDACi at 1, 7, 21, or 48 h after treatment, and plasma samples were assayed. The quantifications of regorafenib and JAK-HDACi in plasma were conducted using a Shimadzu LC-20AD LC (Shimadzu, OR) and an API5000 MS (Sciex, MA). For sample preparation, a one-step protein precipitation with acetonitrile was used. Plasma (10 µL) was added into 250 µL of acetonitrile. Chromatographic separation was performed using an ACE Excel 3 C18 column. The mobile phase was (A) 0.1% formic acid in water and (B) 0.1% formic acid in acetonitrile utilizing a gradient from 20% to 75%. The injection volume was 10 µL. The run time was 5 min, and the retention time was 2.4 min. Standard curves for regorafenib and JAK-HDACi in plasma were prepared in the ranges of 5–15,000 ng/ml.

### Statistical analysis

Data were presented as means ± SD for replicates. Number of each replicate (n) are mentioned in figure legends. Student t test (one-tailed, unequal variance) was used to perform comparisons between the groups, as described in figure legends. *P*-values ≤ 0.05 were considered statistically significant. Combination index (CI) values (for synergy), in in vivo models for JAK/HDACi and regorafenib were calculated by Bliss-independent equations as described by Liu et al., [38].

## Results

### Combination of regorafenib with the dual JAK/HDAC inhibitor reduces CRC cell proliferation

**Fig. 1:**
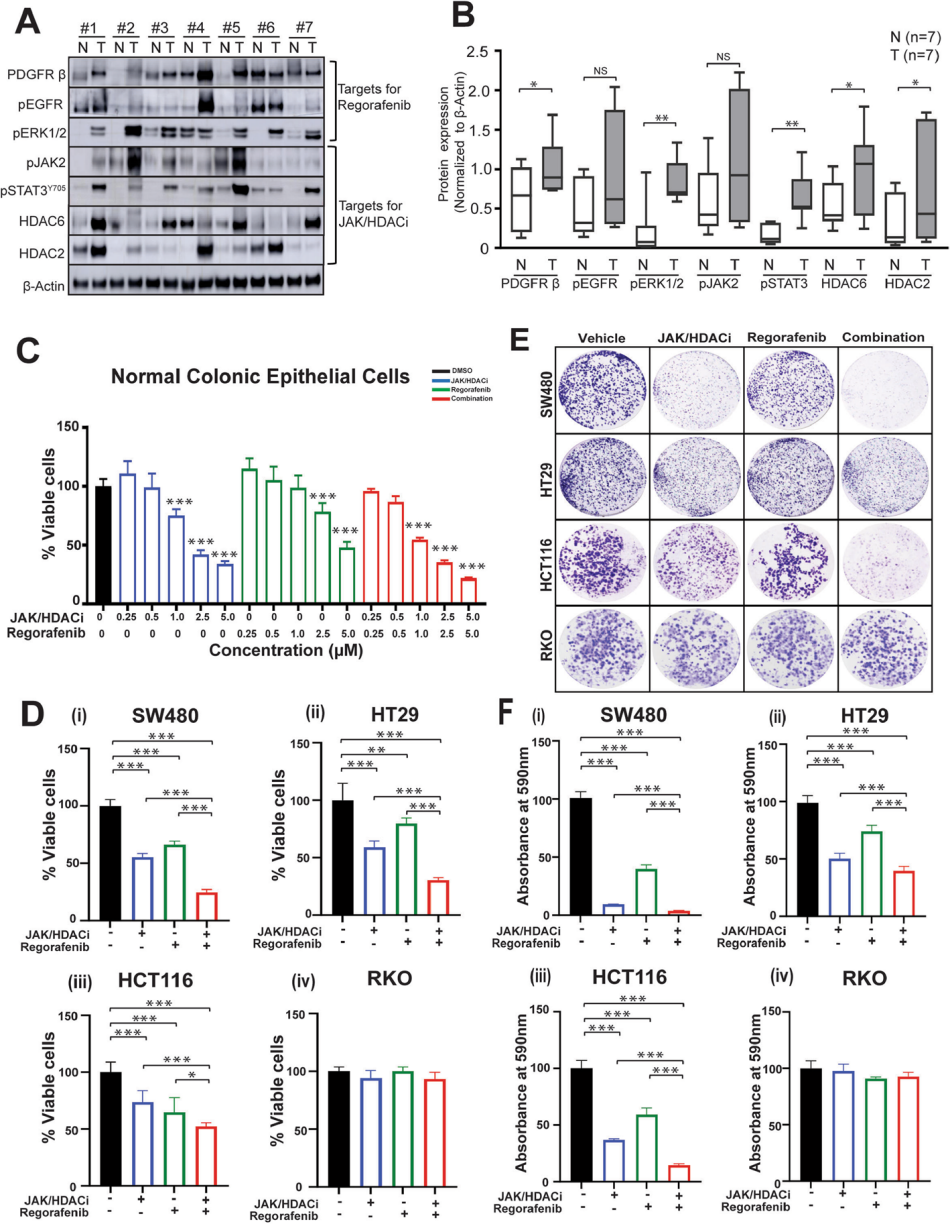
The JAK/HDACi and regorafenib combination reduces cell proliferation and colony formation of CRC cells.** A** Western blotting demonstrating the key targets of JAK/HDACi and regorafenib in tissue lysates of CRCs (T) along with their adjacent, uninvolved normal tissues (N). **B **Figure represents relative protein expression (normalized to their respective β-actin) of drug targets illustrated in Fig A. ** C** MTT assay analyzing the effect on cell proliferation of JAK/HDACi (0.25 – 5.0 µM) and regorafenib (0.25 – 5.0 µM) in single and their combination drug treatments in normal primary colonic epithelium cells. Results are presented as percents of vehicle control (DMSO). **D** MTT assay evaluating the efficacy of JAK/HDACi (500nM) and regorafenib (500nM) alone and in combination drug treatments on proliferation of CRC cell lines. Results are presented as percents of vehicle control (DMSO) in each set (i) SW480, (ii) HT29, (iii) HCT116, and (iv) RKO. **E** Colony assay for four CRC cell lines (SW480, HT29, HCT116, and RKO) showing the efficacy on colony formation of JAK/HDACi (500nM) and regorafenib (500nM) alone and in combination drug treatments. **F** Quantification of colonies was performed by a 10% acetic acid extraction method. Results are presented as averages of triplicates from each treatment group and as percents of vehicle control (DMSO) (i) SW480, (ii) HT29, (iii) HCT116, and (iv) RKO. *P*-values were labeled as follows: * *p* ≤ 0.05, ** *p* ≤ 0.01, and *** *p* ≤ 0.001

Regorafenib inhibits multiple kinases which are activated in key signaling pathways, such as stromal RTKs (FGFRs, PDGFRβ), angiogenic receptor tyrosine kinases (EGFR), and intracellular signaling kinase (ERK1/2) [17]. Several studies [39], have evaluated the association of increased HDACs expression with CRC, additionally inhibition of activated JAK/STAT pathway holds good promise in CRC regression [40]. We therefore used a hybrid molecule JAK/HDACi in combination with regorafenib in our study, with a rationale to inhibit multiple pathways which are activated in CRCs. We first as proof of concept evaluated expression of JAK/HDACi targets (pJAK2, pSTAT3^Y705^, and HDAC2/6) and regorafenib targets (PDGFRβ, pEGFR, and pERK1/2) by western blots in tissue lysates of CRCs (T) and their corresponding normal/uninvolved tissues (N), wherein increased expression was observed in tumor tissue compared to their adjacent normal (Fig. 1A-B), indicating that targeting CRC with this combination could be promising. Since regorafenib toxicity is an issue to be addressed, we evaluated cell proliferation in normal primary colonic cells at varying concentrations (250 nM- 5 µM) of JAK/HDACi and regorafenib individually and in combination and found that, at 500 nM, these normal cells, were unaffected (Fig. 1C). Thus, this concentration was used to assess the effect of drugs individually and in combination on cell proliferation employing four CRC cell lines, i.e., HCT116, RKO, HT29, and SW480, which exhibit different molecular backgrounds (p53, KRAS, BRAF, PIK3CA, JAK, STAT, HDAC mutations, and MSI status) summarized in Table S1. With individual and combination treatments, there was reduced proliferation of SW480, HT29, and HCT116 cells (Fig. 1Di-iii), the effect was pronounced in SW480 and HT29 CRC cells (Fig. 1Di-ii), which exhibit no genetic alterations in JAK/HDAC target molecules (see Table S1). At this dose the effect on cell viability was not noted in RKO cells (Fig. 1Div) At 500 nM, there was a similar pattern of response for colony formation in all four cell lines (Fig. 1E). Significant reductions in the number of colonies were noted for SW480, HT29, and HCT116 cells (Fig. 1Fi-iii), but marginal response was evident for RKO cells (Fig. 1Fiv). HCT116 cells exhibit mutations in all HDACs and JAK1 but are wild-type for STAT3, whereas RKO cells harbor mutations in STAT3, FGFR1, and HDAC6 (see Table S1). These results demonstrate, for CRC cell lines, the efficacy of the combination treatment in the nanomolar range compared to previous studies with higher treatment concentrations of regorafenib [41]. There was a marginal effect on cell viability of normal primary colonic cells at 500 nM concentration in combination treatment (Fig. 1C). At this concentration, the combination treatment in SW480, HT29, and HCT116 has demonstrated a significant decrease in cell viability and colony formation but these effects were not observed in RKO cells. The differential response on cell proliferation may be due to the molecular/genetic background of CRC cells.

### Combination treatment increases cell cycle arrest at the G0-G1 phase and induces apoptosis

**Fig. 2:**
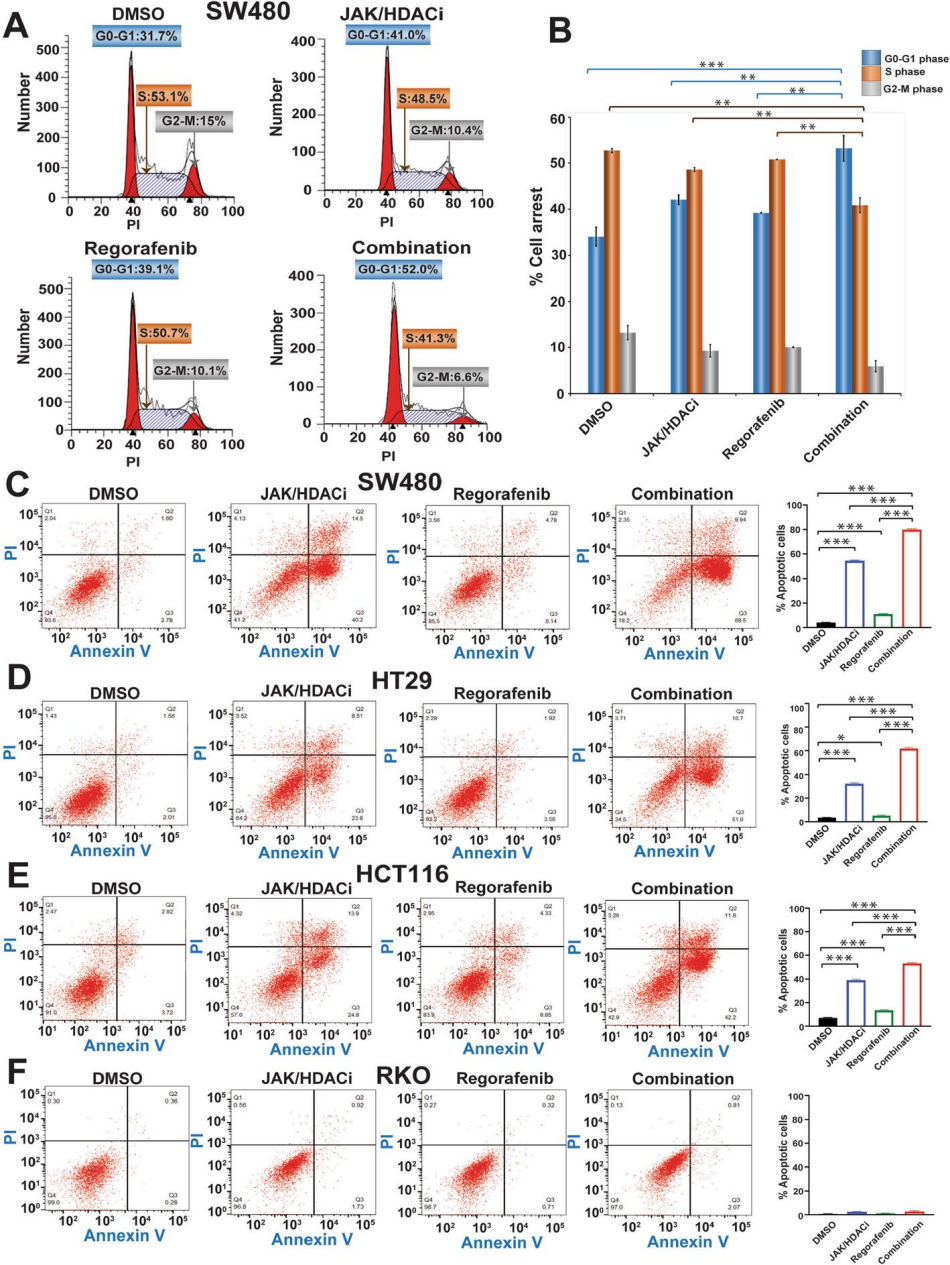
The JAK/HDACi and regorafenib combination induces cell arrest at the G0-G1 phase, delays the S phase, and induces apoptosis. **A** Cell cycle arrest was analyzed by flow cytometry of SW480 cells exposed to DMSO (control), JAK/HDACi, regorafenib, or the combination. **B** The average of three experiments was quantified for each treatment group and plotted as percent cell arrest. **C-F** Apoptosis was analyzed by flow cytometry quantifying FITC-conjugated annexin and propidium iodide (PI) positive cells. Average of three experiments (sum of % early and late apoptosis) were quantified for each treatment group and plotted as percent apoptotic cells. **C** SW480 cells, **D** HT29 cells,** E** HCT116 cells, **F** RKO cells. *P*-values were labeled as follows: * *p* ≤ 0.05, ** *p* ≤ 0.01, and *** *p* ≤ 0.001

To assess the role of these drugs, individually and in combination treatment, on the cell cycle, we conducted experiments with SW480 and RKO cells. For SW480 cells, there were higher numbers of cells arrested at the G0-G1 phase by the combination treatment (52%) as compared to DMSO (31.7%), JAK/HDACi (41%), and regorafenib (39.1%). There was also a delay in the synthesis (S) phase for the combination treatment (41%), as compared to DMSO (51%), JAK/HDACi (48%), and regorafenib (50%) (Fig. 2A-B). However, for RKO cells, there was no change in cell arrest with either individually or with combination treatment (Supplementary Fig. S1A-B). Additionally, in four CRC cell lines, we analyzed the effect of drug treatments on apoptosis by propidium iodide (PI) and annexin V staining. In SW480 cells, there was increased apoptosis in the combination treatment (Fig. 2C), HT29 (Fig. 2D) and HCT116 (Fig. 2E), cells but not observed in RKO cells (Fig. 2F). These results correlate with cell proliferation and colony formation results. For each cell line, quantification is presented as an average of three experiments and as the percentage of apoptotic cells, which includes the sum of early and late apoptotic cells from each treatment group. For SW480 cells, the percentages of apoptotic cells were, for combination treatment (79.91%), JAK/HDACi (54.46%), regorafenib (11.17%), and DMSO (4.04%) (Fig. 2C). For HT29 cells, the percentages of apoptotic cells were, for combination treatment (61.83%), JAK/HDACi (32.28%), regorafenib (5.16%), and DMSO (3.51%) (Fig. 2D). For HCT116 cells, the percentages of apoptotic cells were, for combination treatment (53.03%), JAK/HDACi (39.13%), regorafenib (13.62%), and DMSO (7.04%) (Fig. 2E). For RKO cells, the percentages of apoptotic cells were, for combination treatment (2.8%), JAK/HDACi (2.44%), regorafenib (1.18%), and DMSO (0.73%) (Fig. 2F). These results show that the combination treatment causes G0-G1 arrest, delays the S phase, and induces apoptosis.

### Combination treatment inactivates kinases, inhibits deacetylation of key histone proteins, and modulates DNA accessibility

**Fig. 3:**
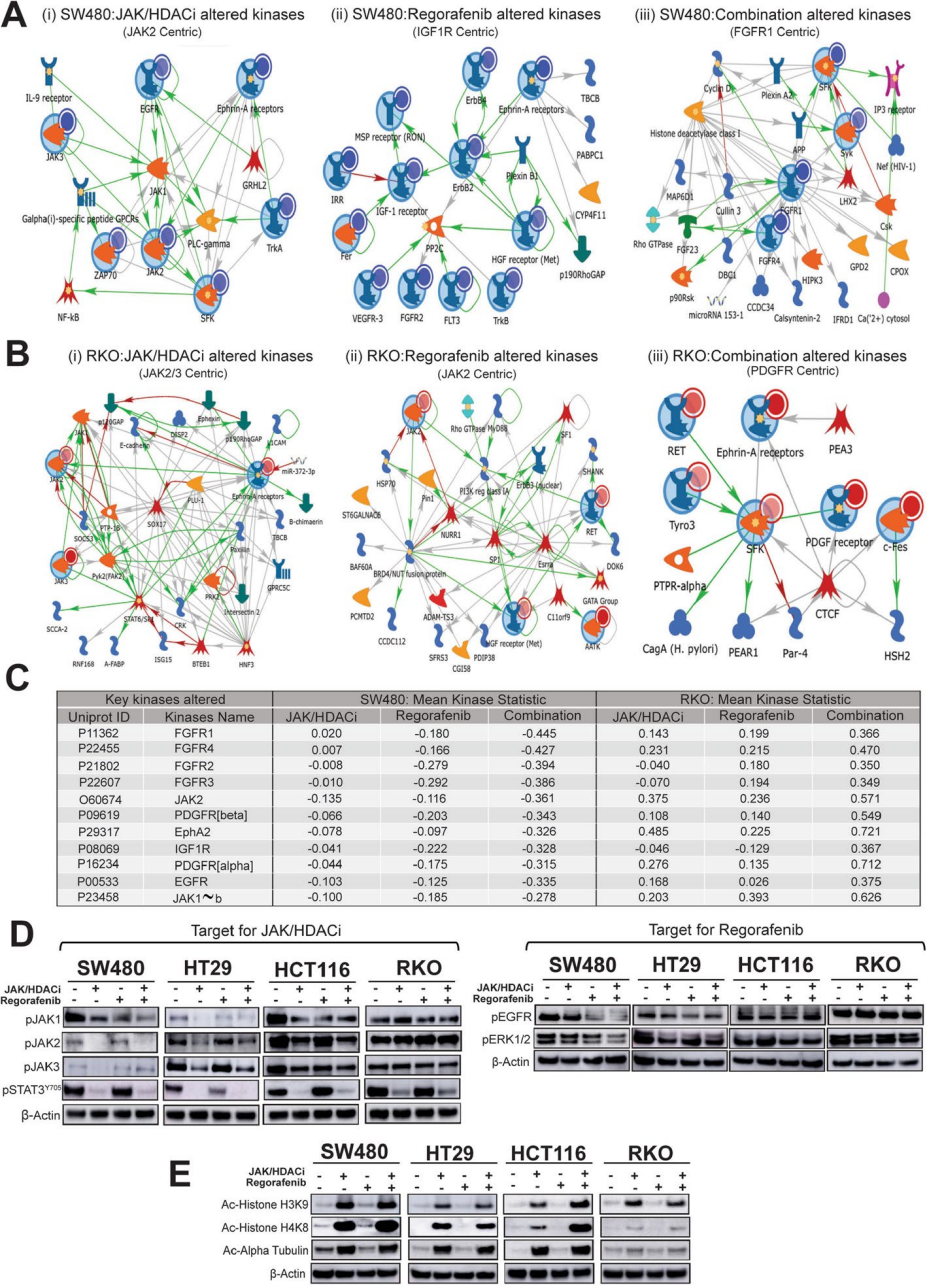
Combination treatment of JAK/HDACi and regorafenib decreases kinase activity in CRC cells. **A-B** Network models of altered kinase activity is displayed. **A** (i) JAK/HDACi, (ii) Regorafenib, and (iii) Combination altered kinases in SW480 cells. **B** (i) JAK/HDACi, (ii) Regorafenib, and (iii) Combination, altered kinases in RKO cells. Networks were generated using MetaCore (portal.genego.com) with an auto expand network set with 75 maximum nodes, with metabolites, and orphan nodes were excluded. Input nodes (kinases) have large blue circles around them. The smaller blue circles represent that kinase activity decreased, while smaller red circles represent that kinase activity increased/unaltered. Arrowheads denote the direction of interactions, and color of the lines indicates type of interaction (green; positive, red; inhibitory, grey; complex). The key for symbols is provided in Table S5. **C** Table summarizes Mean Kinase Statistics (MKS) of kinases modulated by treatment of JAK/HDACi and regorafenib and their combination in SW480 and RKO cells. Mean Kinase Statistic for each treatment groups values are represented as change from DMSO control. **D** Western blotting demonstrating inactivation of kinases. In lysates of four CRC cell lines, JAK/HDACi targets pJAK1, pJAK2, pJAK3, and pSTAT3^Y705^, and regorafenib targets pEGFR and pERK 1/2. β-Actin was used as a loading control. **E** Western blots demonstrating that JAK/HDACi and the combination treatment inhibit deacetylation of histone H3K9, H4K8 and alpha tubulin, as illustrated by increased proteins of acetylated-histone H3K9, H4K8, and α-tubulin. β-Actin was used as a loading control. *P*-values were labeled as follows: * *p* ≤ 0.05, ** *p* ≤ 0.01, and *** *p* ≤ 0.001

Since regorafenib is a multiple-kinase inhibitor [17], and JAK-HDACi also inhibits important kinases [25], we conducted kinome analysis on lysates of SW480 and RKO cells to derive a holistic picture of kinases altered by individual and combination treatment. The rationale to select SW480 and RKO cells was to gain a better understanding of modulation in kinase activities, as these cell lines exhibited distinct responses to treatments (SW480-responsive, RKO-nonresponsive/partial responsive). SW480 cells treated with JAK-HDACi showed decrease in the activity of various kinases, including, JAK2, Ephrin A receptors, EGFR, and others in a signaling network that was JAK2-centric (Fig. 3Ai). SW480 cells treated with regorafenib showed decrease in the kinase activity of Met, VEGFR-3, ErbB4, ErbB2, FLT3, and other receptor tyrosine kinases, and mapped to a network that was IGF1R-centric (Fig. 3Aii). Marked decreases in kinase activity, including SYK, SFK, FGFR1, and FGFR4, were evident after combination treatment, as compared to individual drug treatments (Fig. 3Aiii). These altered kinases mapped to an FGFR1 centric network (Fig. 3Aiii). However, for RKO cells, several kinases were unaltered/activated by drug treatments (Fig. 3Bi-iii).

The inhibition of established target kinases for JAK/HDACi (JAK1/2 and FLT3/4) and regorafenib (FGFR 1–4, EGFR, Src, Fgr, and PDGFRα/β) are shown in Fig. 3C. Additionally, inhibition of unreported kinases for these drugs, which included EphA 1–5, and 8, HER 2–4, and FAK 1–2, was more pronounced in the combination treated SW480 cells as compared to JAK/HDACi or regorafenib alone, as evidenced by reduced kinase activity (Fig. 3C and supplemental Table S4). However, these kinases were not inhibited in RKO cells (Fig. 3C, supplemental Table S4). For four CRC cell lines, we validated the kinome results by western blots, wherein combination treatment showed marked reductions in phosphorylation of JAK1/2/3 and STAT3 (targets for JAK/HDACi) and pEGFR and pERK1/2 kinases (targets for regorafenib) (Fig. 3D). Among the four CRC cell lines, the response to combination treatment differed at varying levels; the higher response was observed for SW480, HT29, and HCT116 cells, and the least response was observed for RKO cells (Fig. 3D).

Cancer progression is governed by genetic mutations and interplay of epigenetic alterations, wherein histones have been considered as potential biomarkers to predict prognosis and patient outcomes [42]. Since ATAC-seq illustrates the chromatin accessibility (hyper-accessible/open or hypo-accessible/closed) of transcription machinery [43, 44], we performed this technique to identify changes in accessible chromatin regions in responsive (SW480) and non-responsive (RKO) CRC cell lines. Our findings have demonstrated striking differences in the patterns of accessible chromatin to regulate the transcription machinery and this change was more pronounced in the combination treatment, specifically in SW480 cells (Supplementary Fig. 2A). For SW480 cells, the open and closed chromatin regions in JAK/HDACi vs. DMSO (hyper-accessible regions: 10,298; hypo-accessible regions: 4932), regorafenib vs. DMSO (hyper-accessible regions: 1408; hypo-accessible regions: 1552), and combination vs. DMSO (hyper-accessible regions: 10,405; hypo-accessible regions: 5137) as shown in Supplementary Fig. S2A. However, RKO was least responsive to drug treatment, which reflects modest changes in accessible chromatin landscape as determined by ATAC-seq. The values were JAK/HDACi vs. DMSO (hyper-accessible regions: 2719; hypo-accessible regions: 906), regorafenib vs. DMSO (hyper-accessible regions: 277; hypo-accessible regions: 419), and combination vs. DMSO (hyper-accessible regions: 2925; hypo-accessible regions: 1253) (Supplementary Fig. S2B).

**Fig. 4:**
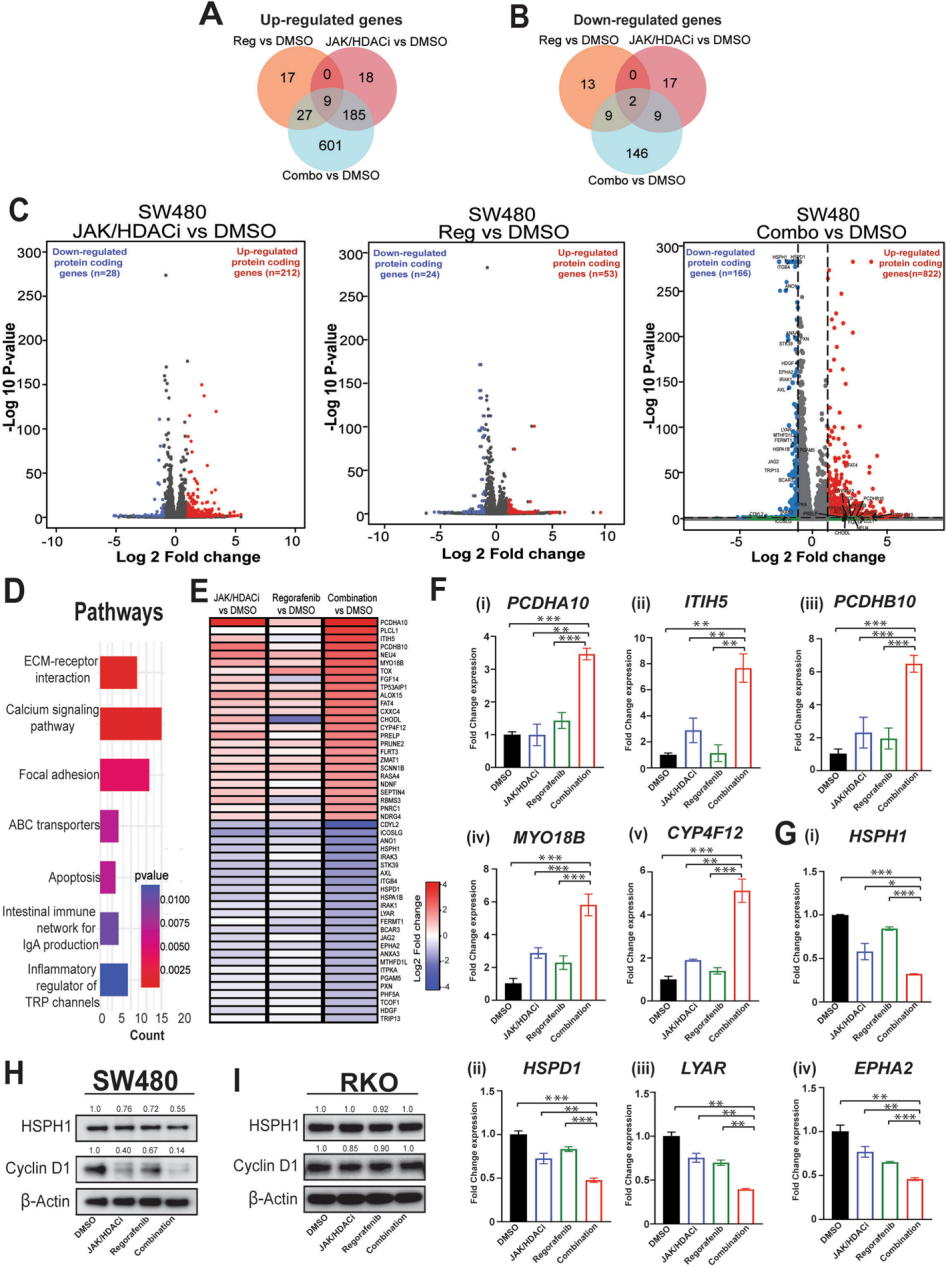
Whole transcriptomic analysis shows that combination treatment of JAK/HDACi and regorafenib modulates important pathways. Results are presented from of duplicates (*n* = 2) from each treatment group. **A**,** B** Venn diagram illustrating number of genes up-regulated/down-regulated by JAK/HDACi (500 nM), regorafenib (500 nM), and in their combination treatment. **C **Volcano plots illustrating statistically significant genes in three comparisons (i) JAK/HDACi (JH) vs. DMSO, (ii) regorafenib (Reg) vs. DMSO, and (iii) combination (combo) vs. DMSO, for SW480 cells. **D** significantly enriched KEGG pathways in genes uniquely altered by combination treatment. **E** Heatmap of genes uniquely modulated in combination treatment. Genes that are differentially expressed with log2 fold change ≥ 1 or ≤ -1 and *p*-value < 0.05 in only combination treatment are referred as uniquely modulated genes. **F**,** G** Quantitative PCR showing expression of gene targets (normalized to GAPDH). **F** The upregulated gene targets are shown (i) *PCDHA10,* (ii) *ITIH5,* (iii) *PCDHB10,* (iv) *MYO18B,* and *(*iv) *CYP4F12,* and **G** the down-regulated gene targets are shown (i) *HSPH1* (ii) *HSPD1* (iii) *LYAR* and (iv) *EPHA2.* **H**,** I** Western blots showing HSPH1 and cyclin D1 protein expression in cell lysates of **H** SW480, and **I** RKO cells. β-Actin was used as a loading control. The values above western blots represent protein expression (normalized to their respective β-actin) compared to their DMSO controls. *P*-values were labeled as follows: * *p* ≤ 0.05, ** *p* ≤ 0.01, and *** *p* ≤ 0.001

The results shown in Supplementary Figs. 2A-B demonstrate the effect of chromatin modulation on gene transcription by drug treatments in SW480 and RKO cells. In SW480 cells, Integrated Genome Viewer (IGV) plots used for data visualization, shows an increase in hyper-accessible chromatin in the transcriptional start-site (TSS) of *CYP4F12* (Supplementary Fig. 2C), resulting in its increased gene expression (Fig. 4E). Since overexpression of CYP4F12 is involved in inhibition of tumor progression and metastasis and its low expression has been associated with poor patient survival [45]; increased expression of *CYP4F12* in the combination therapy demonstrates its therapeutic advantage. Similarly, hypo-accessible chromatin in the TSS of *ITFB4* (Supplementary Fig. 2D) led to reduced gene expression (Fig. 4E). Increased *ITGB4* expression is involved with tumor growth, progression, and metastasis in CRC, and unfavorable overall survival [46]. Thus, decrease in *ITGB4* expression observed in our study indicate the efficacy of combination treatment.

Since HDACs, tumor-promoting enzymes causing deacetylation of acetyl-lysine (KAc) residues on their substrates (histones and non-histone proteins), have emerged as candidates for epigenetic effects, we evaluated how JAK/HDACi, regorafenib, and their combination modulate acetylation of histones. Combination treatment increased intracellular levels of acetyl-histone H4 (Ac-HH4) and acetyl-α-tubulin (Ac-Tub), which are substrates for HDAC1/2/3 and HDAC6, respectively, more dramatically in SW480, HT29, HCT116 as compared to RKO (Fig. 3E).

These results indicate that treatment inhibits drug targets and their respected over-activated kinase pathways, more predominately by combination treatment than by JAK/HDACi and regorafenib agents alone, thus illustrating the higher efficacy of combination treatment. The effect can vary, however, depending on the mutational background of CRC cells.

### Combination treatment modulates signaling pathways

Our transcriptomic profiling results with SW480 cells show that combination treatment up-regulated 601 genes, and down-regulated 146 genes as shown in a Venn diagram (Fig. 4A-B). Volcano plots highlighted the difference in gene expression for individual and combination treatment (Fig. 4C), for which there were a higher number of genes modulated by treatment with the combination. Upregulated protein-coding genes in JAK/HDACi vs. DMSO, regorafenib vs. DMSO, and the combination vs. DMSO were 212, 53, and 822, respectively and downregulated protein-coding genes were 28, 24, and 166, respectively (Fig. 4C).

We further investigated which pathways are altered by these modulated genes in combination treatment. Using Kyoto Encyclopedia of Genes and Genomes (KEGG), which groups genes to similar biological processes at the cellular level, we found that the extracellular matrix-receptor interaction, focal adhesion, and apoptosis pathways were modulated (Fig. 4D), and the representative genes are shown in the heat map (Fig. 4E). Interestingly, increased expression of several tumor suppressor genes (*PCDHA10, ITIH5, PCDHB10*, and *MYO18B*) [47–51], and *CYP4F12* gene [45] involved in inhibition of cell migration and epithelial-mesenchymal transition (EMT) was observed (Fig. 4Fi-v). In addition, we found genes associated with tumor progression were downregulated; among these were heat shock proteins (HSP), such as *HSPH1, HSPD1, LYAR* and *EPHA2* [52–54]. We further validated these findings by performing qPCR (Fig. 4Gi-iv). These genes are well-established in modulating tumor growth and metastasis.

HSPH1 is associated with CRC progression and is involved in regulation of Wnt/β-catenin pathway. Inhibition of HSPH1 inhibits expression of STAT3 and its downstream targets, including cyclin D1 [55]. We verified decreased expression of *HSPH1* observed in transcriptomic profiling further by western blotting and found decrease in HSPH1 protein levels with concomitant reduction in cyclin D1 expression in combination treatment of SW480 cells (Fig. 4H), which was not evident in least responsive RKO cells (Fig. 4I). These results indicate the advantage of combination treatment in differentially modulating the genes involved in key cellular pathways.

### Combination treatment causes a synergistic reduction in CRC PDX growth

**Fig. 5:**
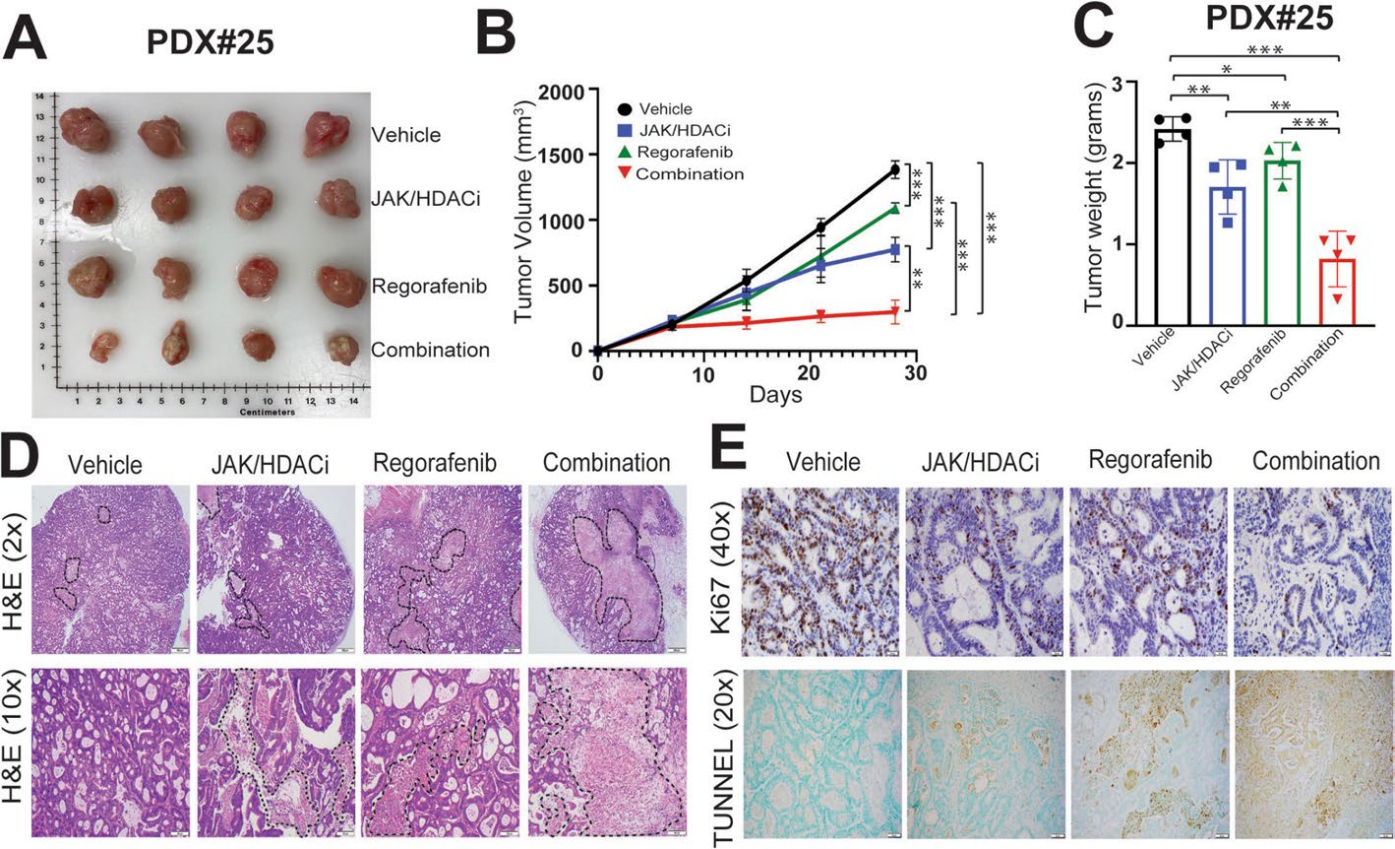
Combination treatment leads to reductions in tumor size, volume, and weight in CRC-PDXs. **A** Image illustrating harvested CRC-PDXs were smaller in the combination treated groups compared to the vehicle control, JAK/HDACi and regorafenib treated groups. **B** Tumor progression kinetics illustrating the efficacy of treatment. The statistical significance is calculated at 28th day of treatment. *P*-values were labeled as follows: * *p* ≤ 0.05, ** *p* ≤ 0.01, and *** *p* ≤ 0.001. **D** H&E staining, illustrating necrotic regions as shown in dotted line.** E** IHC analysis showing decreased numbers of Ki67-positive cells in tissues treated with the combination, indicating fewer proliferating cells (upper panel). Increased TUNEL-positive cells present in the combination-treated group, indicating induced apoptosis (bottom panel)

To evaluate the therapeutic potential of the JAK/HDACi, regorafenib and combination drug treatments in a CRC PDX model, CRC PDX tissues were implanted into flanks of NSG mice. Tumors were routinely measured, and mice were sacrificed after 28 days of treatment. The combination treatment synergistically reduced tumor sizes, tumor volumes, and tumor weights as compared to vehicle control, JAK/HDACi and regorafenib alone (Fig. 5 A-C). Hematoxylin and eosin (H&E) staining of excised tumors showed more necrotic lesions in combination treatment (Fig. 5D). IHC staining showed reduced Ki67-positive staining, indicating that proliferation was reduced by the combination treatment as compared to single drugs and vehicle control treatment groups (Fig. 5E-upper panel). Additionally, combination treatment induced apoptosis as demonstrated by higher numbers of TUNEL-positive cells as compared to individual drug treatments (Fig. 5E-lower panel). These results suggest that combination treatment reduces tumor growth in CRC PDX model by decreasing proliferation and inducing apoptosis.

### Combination treatment reduces experimental metastasis, with no evident toxicity

**Fig. 6:**
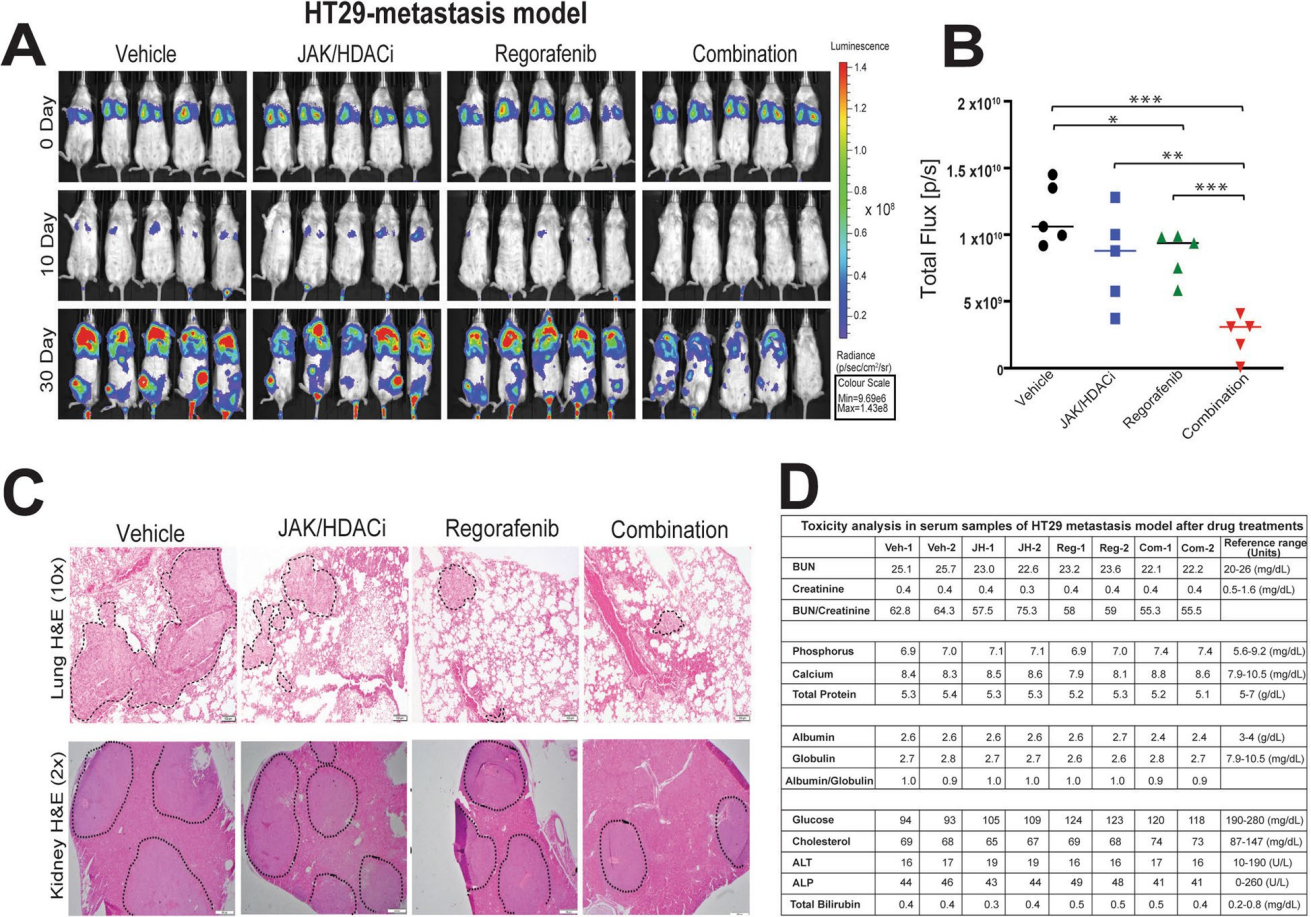
Combination treatment decreases metastasis of luciferase-tagged HT-29 cells injected into NSG mice. **A** Bioluminescence imaging of vehicle- and drug-treated groups at 0, 10, and 30-day intervals, highlighting efficacy of the treatment for the combination group, as evidenced by reduced bioluminescence intensity of luciferase-tagged HT29 cells. **B** Quantification of bioluminescence intensity on day 30th of vehicle and drug-treated groups, showing reduced intensities in the regorafenib- and combination-treated groups. *P*-values were labeled as follows: * *p* ≤ 0.05, ** *p* ≤ 0.01, and *** *p* ≤ 0.001. **C** H&E staining of FFPE sections from lung at 10X magnification (top panel) and kidney at 2X magnification (bottom panel) showing reduced tumor burdens in these organs in combination treatment. **D** Toxicity analysis in serum samples of mice with the vehicle- and drug-treated HT29 metastatic model. Values were within the reference range, illustrating no evident toxicity was observed by either by JAK/HDACi and regorafenib alone or their combination treatment

We evaluated the effects of individual and combination drug treatment on metastasis using an HT29 experimental model. There was significant reduction in the distant spread of HT29 luciferase tagged cells as shown by non-invasive bioluminescence imaging in combination treatment (Fig. 6A). The bioluminescence intensity was quantified as total flux on the 30th day of treatment. Efficacy of the combination treatment was reflected by reduced luciferase activity as compared to vehicle and individual drug treatments (Fig. 6B). H&E staining of lung and kidney sections corroborated well with bioluminescence imaging of smaller tumors (shown with dotted lines), indicating fewer metastases to distant organs (Fig. 6C). To evaluate the effect of drug treatment on kidney and liver functions, serum analysis was performed on the last day of the experiment. All parameters were within the reference range, indicating no signs of toxicity after drug administration (Fig. 6D). These results suggest that combination treatment decreases metastasis of luciferase-tagged HT29 cells, and has no evident toxicity.

### The antitumor immune response is enhanced by the combination treatment showing increased infiltration of CD45 and cytotoxic CD8 T cells

**Fig. 7:**
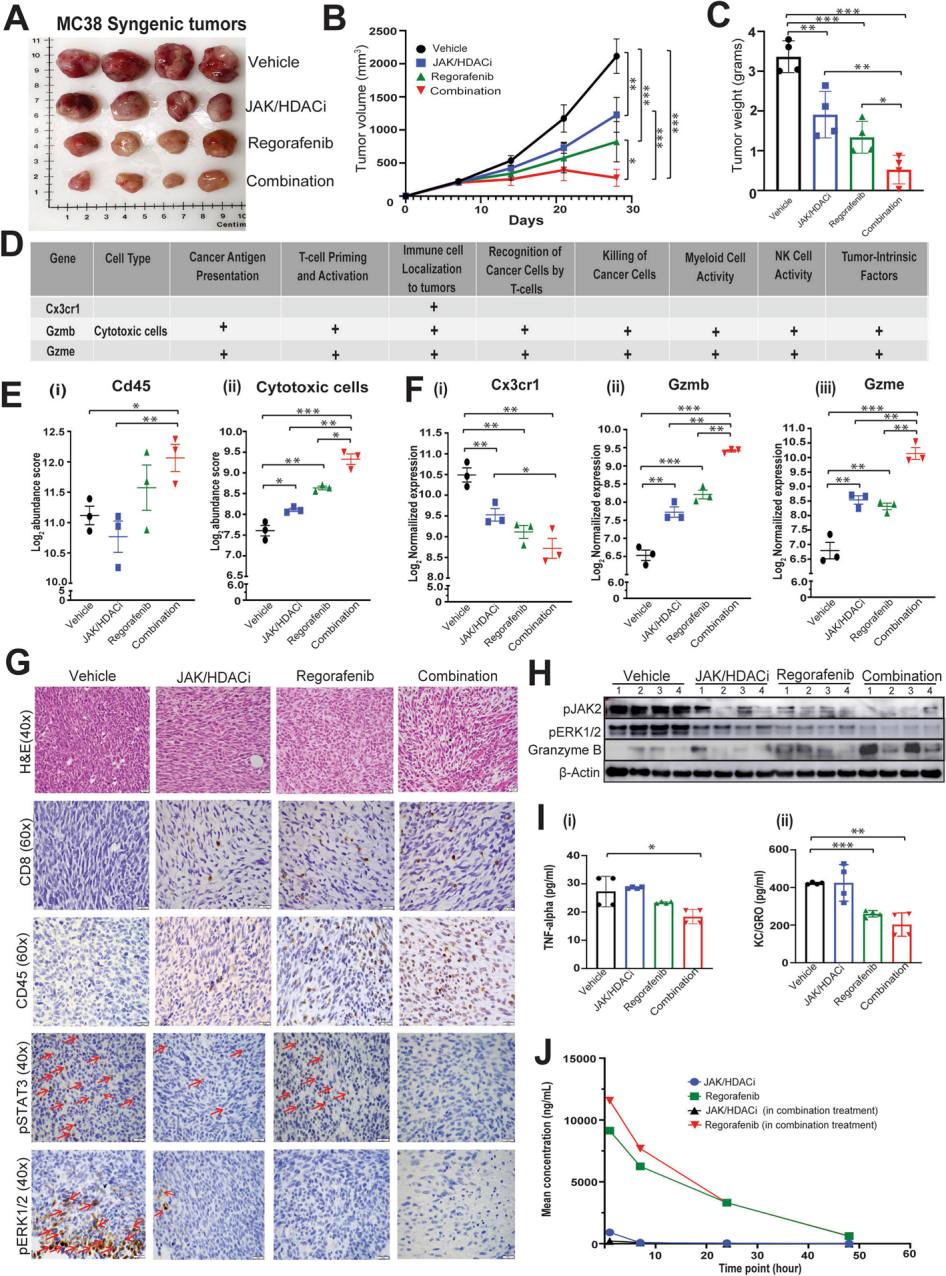
Combination treatment reduces tumor growth in an MC38 syngeneic mouse model, and increased infiltration of CD45 and cytotoxic CD8 T cells. **A** Image illustrating that harvested syngeneic tumors were smaller in treated groups compared to vehicle control **B** Tumor progression curve illustrating efficacy of the treatment; in the combination-treated group the tumors regressed, and statistical significance is calculated at 28th day of treatment. *P*-values were labeled as follows: * p ≤ 0.05, ** p ≤ 0.01, and *** p ≤ 0.001. **C** Tumor weights (in grams) were reduced in the combination-treated group. **D** Table summarizing the functional aspects of modulated genes in the combination-treated group. **E** Higher Log_2_ abundance scores of Cd45 and cytotoxic cells were evident for the combination-treated group. **F** Gene expression of targets (i) *Cx3cr1*, (ii) *Gzmb*, and (iii) *Gzme* were altered in JAK/HDACi, regorafenib and the combination-treated group. **G** H&E and IHC staining for CD8, CD45, pSTAT3^Y705^ and pERK1/2 are shown. **H** Western blots illustrating expressions of pJAK2, pERK1/2, and granzyme B. **I** Plasma analysis for cytokines released after drug treatment (i)TNF-Alpha and (ii) KC/GRO. **J** Pharmacokinetics in plasma samples of C57BL/6 mice after JAK/HDACi and regorafenib alone and in their combination treatment at 1, 7, 21, and 48 h

To evaluate the effect of drug treatment on immunomodulation, we employed a syngeneic mouse model. Murine CRC MC38 cells were injected into flanks of C57BL/6 mice and were treated with test agents alone or in combination every third day. The combination treatment synergistically reduced tumor sizes (Fig. 7A), tumor progressions (Fig. 7B), and tumor weights (Fig. 7C), as compared to vehicle control and individual treatments of JAK/HDACi and regorafenib (Fig. 7 A-C). To investigate the association of drug treatment on the immune response, we analyzed gene expression using the Immuno Oncology (IO360 Mouse) panel of Nanostring (Fig. 7D). and found that the combination treatment had higher *Cd45* and cytotoxic cells abundance scores (Fig. 7Ei-ii). Interestingly, we observed marked reduction in CX3C motif chemokine receptor 1(*Cx3cr1*) and significant increased expression of *Gzmb* an *Gzme* in combination treatment (Fig. 7Fi-iii). We confirmed the finding with IHC staining of FFPE sections of harvested tumors. There was higher CD45 staining in tissue sections following combination treatment, corroborating Nanostring gene expression results. CD45 has phosphatase activity and dephosphorylates JAK2 [56], which modules STAT3 [56, 57]; this finding corroborates with our results, where we observed, decreased pJAK2 (Fig. 7H), and decreased pSTAT3^Y705^ expression (Fig. 7G) in the combination treatment. Thus, concomitant increased CD45 expression (Fig. 7E(i) and G), corelating well with inactivation of pJAK2 and pSTAT3 ^Y705^ levels. Serum analysis showed the inflammatory cytokines, tumor necrosis factor alpha (TNFα) and keratinocyte chemoattractant/human growth-regulated oncogene KC/GRO, were reduced after treatment with the combination (Fig. 7Ii-ii). Previous study [58] has reported regorafenib induces the immune response by CD8 infiltration, interestingly our results show pronounced infiltration of CD8 T cells, after combination treatment, relative to regorafenib alone (Fig. 7G). The results suggest that, therefore combination treatment increases immune response of regorafenib. Further, to evaluate the bioavailability of JAK/HDACi, regorafenib, and their combination, plasma samples from C57BL/6 mice were used for pharmacokinetic analysis. When given in combination with JAK/HDACi, the bioavailability of regorafenib increased, indicating the efficacy of the treatment could be due prolonged bioavailability (Fig. 7J). In sum, our results demonstrate that the combination treatment enhances antitumor immunity.

## Discussion

Our study evaluated combination therapy in reducing the drug-induced toxicity by regorafenib, a multi-kinase inhibitor used to treat mCRC patients, refractory to first-line FU- or irinotecan-based therapy [59]. We assessed the efficacy of a combination, JAK/HDACi with regorafenib at nanomolar concentrations, to reduce the drug-induced toxicity of regorafenib by lowering its dosage and evaluating the efficacy of JAK/HDACi and regorafenib alone and their combination treatments. JAK/HDACi is a dual/hybrid molecule, showing promise in treatment of other cancers [25]. Preclinical studies have evaluated the anticancer activities of HDACi, due to their role in cell cycle arrest and apoptosis [60] HDACi modulate tumor vasculature to reduce angiogenesis and regulate host immune responses [61, 62]. To date, four HDACi, belinostat (PXD101), romidepsin (FK228), panobinostat (LBH589), and vorinostat (SAHA) have been approved by the US Food and Drug Administration (FDA). Although HDACi alone shows promising effects, and the above-mentioned HDACi are in clinical use, these, like regorafenib, have side effects, which include, but are not limited to, fatigue, diarrhea, nausea, vomiting, and cardiotoxicity [61–64]. In addition, they have limited efficacy in treatment of solid tumors [65]. To address these shortcomings, several groups have designed isoform selective HDACi and dual/bifunctional/hybrid/multitarget inhibitors [66]. These hybrid inhibitors modulate various pathways, as several bioactive groups are assembled into one compact molecule, thereby circumventing pharmacokinetic limitations [67–69].

One of the drawbacks of SAHA treatment, is activation of the JAK-STAT pathway as reported by Liang et al. [25], by upregulating phosphorylation of STAT3 at Tyr705 residue. However, it is interesting to note, that this JAK/HDACi suppresses the JAK-STAT pathway, as evidenced by reduced phosphorylation of STAT3 at Tyr705 with JAK/HDACi alone and its combination with regorafenib. Additionally increased stabilization of acetylation was observed in Ac-HH3, Ac-HH4 and Ac-Tub, which potentiates the fact, that this inhibitor concurrently inhibits both JAKs and HDACs, and combination with regorafenib is further advantageous as multiple oncogenic kinases are inhibited.

The results of our study demonstrate that the combination of JAK/HDACi and regorafenib, which inhibits key targets that are upregulated in CRC, is promising. Treatment with 500 nM concentration, which has marginal toxicity to normal primary colonic epithelium cells, is effective in reducing CRC cell proliferation, as key targets of both drugs are activated in fast-dividing CRC cells. The inhibition of activated kinases is evidenced by kinome analyses of SW480 cells, which illustrates effectiveness of the drug treatment with JAK/HDACi and regorafenib, and combination drug treatment. The combination therapy showed varied responses for various CRC cell lines, as observed in analyses of cell proliferation, colony formation, inactivation of key kinases, inhibition of kinase activity, cell cycle, and apoptosis.

Cancer cells modulate cellular metabolism and heavily rely on chaperones like HSPH1, as this protein has anti-aggregation properties and can modify the misfolded proteins, and thus cell can sustain cellular stress and survive [55]. HSPH1 has been previously reported to promote cell growth by activation of STAT3 [55]. Thus, dramatic decrease in HSPH1 expression observed by whole transcriptomic analysis in SW480, by combination treatment was a promising observation, which leads to inactivation of STAT3 and decrease in its downstream target cyclin D1. We speculate that the partial response for RKO cells could be due to its constitutively activated STAT3, since RKO cells harbor a p.E616del mutation, present in the SH2 domain of the protein. OncoKB annotates [70] that the STAT3 E616del alteration as likely oncogenic [71]. It is a gain-of-function variant [72], as STAT3 activation is accomplished without a stimulus, it is due to higher positive electrostatic potential, which increases interaction with the DNA phosphate carrying a negative charge. This electrostatic interaction increases the binding affinity to DNA and leads to increased transcriptional activity of STAT3 and to its longer nuclear retention [72]. Molecular dynamics simulations conducted by Husby et al. [71] showed that E616 residue of STAT3 a key amino acid of monomer-B involved in the STAT3 protein-DNA interaction. Using cBioportal [73], we evaluated 18 studies of colon and rectal cancers with 7162 patients to investigate the mutational frequency of E616del; we found that only 4 patients had this STAT3 mutation. Thus, our findings indicate that treatment with the combination would benefit a wide spectrum of CRC patients.

Our investigation showed promising results for the CRC-PDX model, the luciferase-tagged HT29 metastasis model, and the MC38 syngeneic mouse model. The Nanostring nCounter PanCancer IO 360™ Panel revealed an increase in abundance of *Cd45* and cytotoxic cells. The elevated numbers of CD45 cells after combination treatment is relevant, as CD45 dephosphorylates JAK and negatively regulates JAK-STAT signaling [57]. CD45 also negatively impacts cytokine receptor signaling by suppressing JAK kinases [56]. It acts as protein phosphatase and binds to JAKs. Targeted cd45 disruption, through increased cytokine and interferon-receptor signaling, activates JAKs and STAT [56]. The results from our syngeneic mouse model demonstrate that, higher the number of infiltrating CD45 lymphocytes, more pronounced is the dephosphorylation of JAK2, which is in corroboration with Sasaki et al. [56] findings, illustrating dephosphorylation of JAK2 is due to protein-tyrosine phosphatase activity of CD45. Further, there are increased abundance scores of cytotoxic cells, which are reflected by elevated *Gzmb* and *Gzme* expression, further demonstrating the treatment efficacy. We observed reduced expression of *Cx3cr1*, in treatment groups compared to the vehicle control; the effect was more pronounced for the combination treatment. These results are in line with the suggestion [74] that CX3CR1 is involved in the tumor microenvironment by contributing to angiogenic macrophage survival, thus promoting metastasis. Zheng et al. [74] found that, in human colon carcinomas, CX3CR1 is expressed in a stage- and histologic grade-dependent manner, where a poor prognosis was associated with CX3CR1 upregulation in tumor-associated macrophages. Their findings further showed that the metastasis of CRC cells was lowered when CX3CR1 was absent in the tumor microenvironment.

Our studies suggested reduced TNFα levels in serum samples of mice treated with the combination. TNFα, a pro-inflammatory mediator involved in apoptosis, is negatively regulated by CD45 [75]. This is consistent with low TNFα levels, as combination-treated mice had high numbers of CD45-positive cells. Thus, our investigations show that the therapeutic combination enhances the immune response at the tumor site and the marked regression of tumors after combination treatment. Pharmacokinetic studies with C57BL/6 mice showed that, after i.p. administration, the bioavailability of regorafenib increased in the combination treatment compared to single treatments. As reported in the literature, several factors contribute to enhanced bioavailability of the drugs due to beneficial pharmacokinetic interactions, for which one drug alters distribution of another drug by increasing absorption, inhibiting metabolism, and/or decreasing excretion, leading to prolonged drug plasma levels [76–78].

The preclinical findings reported in this study provide a basis to investigate further benefits of combining regorafenib with the JAK/HDACi hybrid molecule. Future studies should focus on a) investigating the role of the tumor microenvironment, including stromal and immune cells, in the combination treatment of the MC38 syngeneic mouse model; b) expanding the preclinical studies to assess the use of immune checkpoint inhibitors by combination of regorafenib with JAK/HDACi; and c) assessing the effect of epigenetic modifications of the genes identified by ATAC-seq.

## Conclusion

In summary, our preclinical study shows efficacy of the combination therapy as compared with JAK/HDACi and regorafenib alone. Moreover, the combination therapy is promising, as it has no evident toxicity. These findings lend support to a clinical trial to assess this combination for treatment of patients with advanced CRC.

### Supplementary Information

Additional file 1: Supplementary Fig. 1 The JAK/HDACi and regorafenib combination does not alter cell cycle in RKO cells. A Cell cycle arrest was analyzed by flow cytometry of RKO cells exposed to DMSO (control), JAK/HDACi, regorafenib, or the combination. B The average of three experiments was quantified for each treatment group and plotted as percent cell arrest. The NS, represent non-significant results. Supplementary Fig. 2 Treatment with the JAK/HDACi and regorafenib combination modulates the chromatin accessibility landscape of SW480 cells more prominently than that of RKO cells. A-B Distribution of differentially accessible regions (DARs) over chromosomes in A SW480 and B RKO cells. The hyper- and hypo-accessible regions are indicated in red and blue colors, respectively. C, D IGV plots demonstrating individual tracks of DMSO, JAK/HDACi, regorafenib and their combination treatment in SW480 and RKO cells. C Hyper-accessible regions (shaded in red) for *CYP4F12* D Hypo-accessible regions (shaded in blue) for *ITGB4*. The TSS and direction of transcription is indicated with blue arrow.

Additional file 2: Supplementary Tables: Table S1. Genes status in CRC cell lines, Table S2. List of antibodies used in this study, Table S3. Primer Sequences for validation of gene targets Table S4. Mean Kinase Statistic in SW480 and RKO cell lines treated with JAK/HDACi and regorafenib and their combination compared their respective control (DMSO treatment), Table S5. Symbol key for network modeling of altered kinases with MetaCore.

## Data Availability

The RNA-seq data provided in this manuscript are publicly available from the NCBI Gene Expression Omnibus (GEO) with accession number GSE252554. Other data generated in this study will be available upon request.

## Abbreviations

ATAC: Assay for transposase-accessible chromatin
ATCC: American type culture collection
CRC: Colorectal cancer
CX3CR1: CX3C motif chemokine receptor 1
EGFR: Epidermal growth factor receptor
ERK: Extracellular signal-regulated kinase
FGFR: Fibroblast growth factor receptor
FU: Fluorouracil
HDAC: Histone deacetylase
HSP: Heat shock proteins
JAK: Janus kinase
KC/GRO: Keratinocyte chemoattractant/human growth-regulated oncogene
mCRCs: Metastatic colorectal cancers
MTT: 3-(4,5-Dimethylthiazol-2-yl)-2,5-diphenyltetrazoliumbromide
NSG: NOD/SCID/IL2γ receptor-null
OS: Overall survival
PDGFR: Platelet-derived growth factor receptor
PDX: Patient-derived xenograft
RTK: Receptor tyrosine kinases
STAT3: Signal transducer and activator of transcription 3
TNF: Tumor necrosis factor
