# Supplementary figures and images for: Combination of dual JAK/HDAC inhibitor with regorafenib synergistically reduces tumor growth, metastasis, and regorafenib-induced toxicity in colorectal cancer
Source: J Exp Clin Cancer Res. 2024 Jul 11;43:192. doi: 10.1186/s13046-024-03106-8 (PMC11238352; doi:10.1186/s13046-024-03106-8)

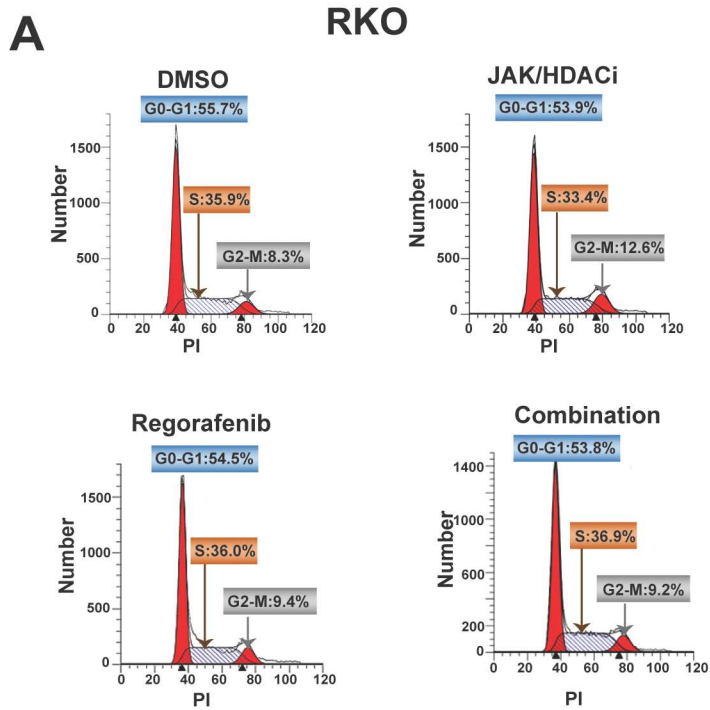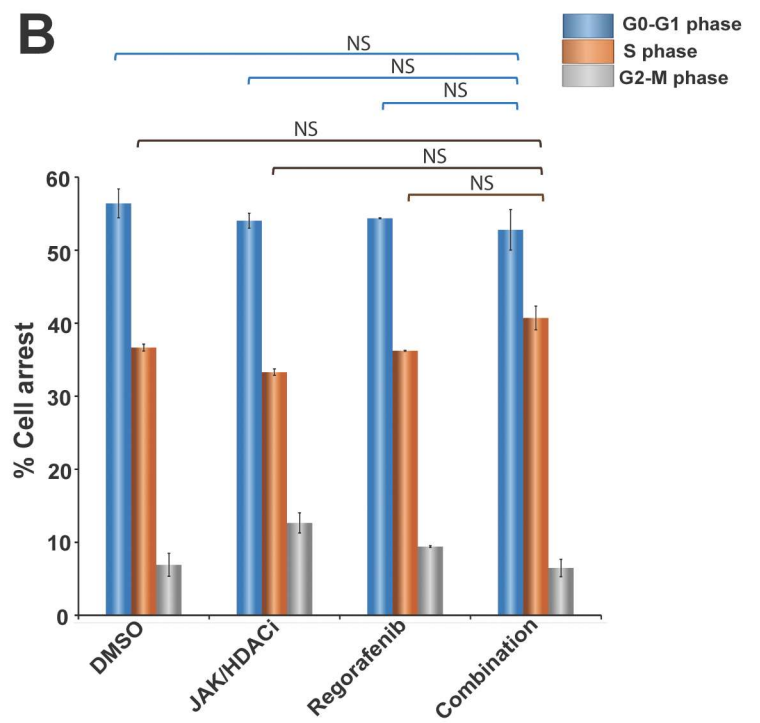

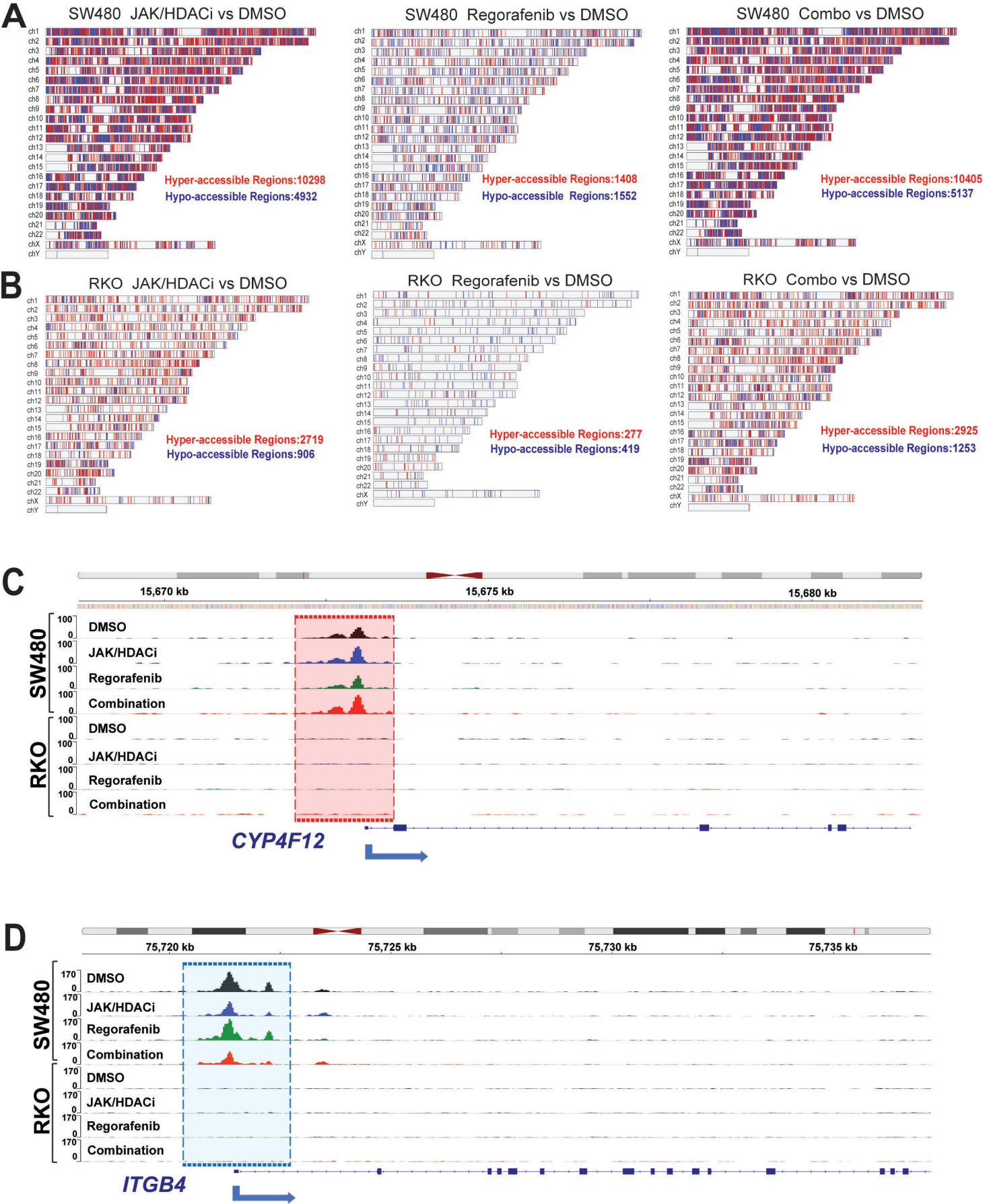

Supplement: Supplementary file 1 — Additional file 1: Supplementary Fig. 1 The JAK/HDACi and regorafenib combination does not alter cell cycle in RKO cells. A Cell cycle arrest was analyzed by flow cytometry of RKO cells exposed to DMSO (control), JAK/HDACi, regorafenib, or the combination. B The average of three experiments was quantified for each treatment group and plotted as percent cell arrest. The NS, represent non-significant results. Supplementary Fig. 2 Treatment with the JAK/HDACi and regorafenib combination modulates the chromatin accessibility landscape of SW480 cells more prominently than that of RKO cells. A-B Distribution of differentially accessible regions (DARs) over chromosomes in A SW480 and B RKO cells. The hyper- and hypo-accessible regions are indicated in red and blue colors, respectively. C, D IGV plots demonstrating individual tracks of DMSO, JAK/HDACi, regorafenib and their combination treatment in SW480 and RKO cells. C Hyper-accessible regions (shaded in red) for CYP4F12 D Hypo-accessible regions (shaded in blue) for ITGB4. The TSS and direction of transcription is indicated with blue arrow. [file 13046_2024_3106_MOESM1_ESM.pdf]
