# Supplementary material for: Combination of dual JAK/HDAC inhibitor with regorafenib synergistically reduces tumor growth, metastasis, and regorafenib-induced toxicity in colorectal cancer
Source: J Exp Clin Cancer Res. 2024 Jul 11;43:192. doi: 10.1186/s13046-024-03106-8 (PMC11238352; doi:10.1186/s13046-024-03106-8)

Table S1. Genes status in CRC cell lines

Gene targets commonly screened	CRC cell lines			
	**HCT116**	**RKO**	**HT29**	**SW480**
**TP53**	WT	WT	p.R273H	p.R273H<br>p.P309S
**KRAS**	p.G13D	WT	WT	p.G12V
**BRAF**	WT	p.V600E	p.V600E<br>p.T119S	WT
**PIK3CA**	p.H1047R	p.H1047R	WT	WT
**PTEN**	WT	WT	WT	WT
**MSI/MSS**	MSI	MSI	MSS	MSS
**Gene targets of the drugs used in this study**	**HCT116**	**RKO**	**HT29**	**SW480**
**STAT3**	WT	p.E616del<br>(in_frame_del)	WT	WT
**HSPH1**	Splice_Site Variant<br>Transcript<br>ENST00000320027.5	WT	WT	WT
**FGFR1**	p.P802P<br>p.D430D<br>p.A343V	p.G608G<br>p.P59L	WT	WT
**HDAC1**	p.S88N	WT	WT	WT
**HDAC2**	p.G395G	WT	WT	WT
**HDAC6**	p.T980T	p.F623F	WT	WT
**HDAC7**	p.P768fs	WT	WT	WT
**HDAC9**	p.Q433fs<br>p.P444P	WT	WT	WT
**JAK1**	p.Y281Y	WT	WT	WT
**JAK2**	WT	WT	WT	WT
**JAK3**	WT	WT	WT	WT

Table S2. List of antibodies used in this study.

Antibody	Application	Dilution	Supplier	Cat. No.
Phospho-Jak1	IB	IB, 1:1000	Cell Signaling Technology, Danvers, MA	4129
Phospho-Jak2	IB	IB, 1:1000	Cell Signaling Technology, Danvers, MA	8082
Phospho-Jak3	IB	IB, 1:1000	Cell Signaling Technology, Danvers, MA	5031
Phospho-STAT3 (Y705)	IB	IB, 1:1000<br>IHC, 1:100	Cell Signaling Technology, Danvers, MA	9145
Phospho-p44/42 MAPK (Erk1/2)	IB	IB, 1:1000<br>IHC, 1:150	Cell Signaling Technology, Danvers, MA	4370
HDAC2	IB	IB, 1:1000	Cell Signaling Technology, Danvers, MA	5113
HDAC6	IB	IB, 1:1000	Cell Signaling Technology, Danvers, MA	7558
PDGF Receptor β	IB	IB, 1:1000	Cell Signaling Technology, Danvers, MA	3129
HSPH1	IB	IB, 1:1000	PTG Labs, Chicago, IL	13383-1-AP
Cyclin D1	IB	IB, 1:1000	PTG Labs, Chicago, IL	26939-1-AP
Ac-Histone H3 (Lys9)	IB	IB, 1:1000	Cell Signaling Technology, Danvers, MA	9649
Ac-Histone H4 (Lys8)	IB	IB, 1:1000	Cell Signaling Technology, Danvers, MA	2594
Acetyl-α-Tubulin (Lys40)	IB	IB, 1:1000	Cell Signaling Technology, Danvers, MA	3971
β –actin	IB	IB, 1:1000	PTG Labs, Chicago, IL	HRP-60008
Granzyme B	IB	IB, 1:1000	PTG Labs, Chicago, IL	13588-1-AP

Ki67	IHC	IHC, 1:200	Abcam, Waltham, MA	ab16667
CD8	IHC	IHC, 1:1500	Abcam, Waltham, MA	ab209775
CD45	IHC	IHC, 1:500	Novus Biologicals, CO	NB100-77417
Anti-Rabbit IgG HRP	IB	IB, 1:5000<br>IHC, 1:1000	Proteintech, Rosemont, IL	SA00001-2
Anti-Mouse IgG HRP	IB	IB, 1:5000	Proteintech, Rosemont, IL	SA00001-1

IB: Immunoblotting; IHC: Immunohistochemical

Table S3. Primer Sequences for validation of gene targets

Gene	Primer sequence 5'-> 3'
PCDHA10-Forward Primer	AGTGCTTGTTCTGCGGAAG
PCDHA10-Reverse Primer	TCTGTCAAAGATAGGGGCGTT
ITIH5-Forward Primer	AGAATGTGGTATTCGTGCTTGAC
ITIH5-Reverse Primer	TCCAATGATACTGAAACGGTCCT
PCDHAB10-Forward Primer	TCACGCGCCAGTATTTCAGG
PCDHAB10-Reverse Primer	CGATGCGTACAGTAGAGGTCC
MYO18B-Forward Primer	CTCAGGACGACCAGTCAAGC
MYO18B-Reverse Primer	GAAGGGGACAGTCTTTTTGGTT
CYP4F12-Forward Primer	GCATCCTGGCTTGGACCTATG
CYP4F12-Reverse Primer	ACATCTGGGTCGAGTTCTTCA
HSPH1-Forward Primer	ACAGCCATGTTGTTGACTAAGC
HSPH1-Reverse Primer	GCATCTAACACAGATCGCCTCT
HSPD1-Forward Primer	GTGTAGACCTTTTAGCCGATGC
HSPD1-Reverse Primer	GTGCCAGTACAGTAGCAGTGG
LYAR-Forward Primer	AACAGCGAACCAGTCAATAAGG
LYAR-Reverse Primer	GGCGTCTTTCACTTTGGAGG
EPHA2-Forward Primer	TGGCTCACACACCCGTATG
EPHA2-Reverse Primer	GTCGCCAGACATCACGTTG
GAPDH-Forward Primer	GGAGCGAGATCCCTCCAAAAT
GAPDH-Reverse Primer	GGCTGTTGTCATACTTCTCATGG

Table S4. Mean Kinase Statistic in SW480 and RKO cell lines treated with single drug and in combination normalized with their respective control (DMSO treatment)

PTK Mean Kinase Statistic							
Kinases		SW480			RKO		
Kinase Uniprot ID	Kinase Name	JAK-HDACi	Reg	combo	JAK-HDACi	Reg	combo
P11362	**FGFR1**	0.020	-0.180	-0.445	0.143	0.199	0.366
P22455	**FGFR4**	0.007	-0.166	-0.427	0.231	0.215	0.470
P12931	**Src**	-0.058	-0.128	-0.376	0.103	-0.068	0.335
P21802	**FGFR2**	-0.008	-0.279	-0.394	-0.040	0.180	0.350
P22607	**FGFR3**	-0.010	-0.292	-0.386	-0.070	0.194	0.349
O60674	**JAK2**	-0.135	-0.116	-0.361	0.375	0.236	0.571
P00519	**Abl**	-0.078	-0.154	-0.349	0.112	-0.036	0.415
P09619	**PDGFR[beta]**	-0.066	-0.203	-0.343	0.108	0.140	0.549
P21860	**HER3**	-0.090	-0.158	-0.345	0.088	0.047	0.476
P54756	**EphA5**	-0.225	-0.324	-0.317	0.094	-0.050	0.343
P29317	**EphA2**	-0.078	-0.097	-0.326	0.485	0.225	0.721
P16234	**PDGFR[alpha]**	-0.044	-0.175	-0.315	0.276	0.135	0.712
P00533	**EGFR**	-0.103	-0.125	-0.335	0.168	0.026	0.375
P30530	**Axl**	-0.082	-0.146	-0.328	0.160	-0.027	0.351
P29320	**EphA3**	-0.066	-0.136	-0.293	0.544	0.028	0.653
Q14289	**FAK2**	-0.038	-0.181	-0.326	0.014	-0.049	0.387
P08069	**IGF1R**	-0.041	-0.222	-0.328	-0.046	-0.129	0.367
Q05397	**FAK1**	-0.053	-0.131	-0.325	0.139	0.052	0.426
P54764	**EphA4**	-0.059	-0.155	-0.268	0.205	-0.122	0.354
P23458	**JAK1~b**	-0.100	-0.185	-0.278	0.203	0.393	0.626
P35916	**FLT4**	-0.012	-0.234	-0.239	0.161	0.133	0.470
P17948	**FLT1**	-0.029	-0.166	-0.273	0.258	0.207	0.573
P21709	**EphA1**	-0.008	-0.125	-0.237	0.425	0.168	0.740
P04626	**HER2**	-0.053	-0.225	-0.292	0.049	0.090	0.456
P29322	**EphA8**	0.022	-0.155	-0.209	0.330	-0.006	0.524
P09769	**Fgr**	-0.045	-0.185	-0.227	-0.246	-0.272	0.229
P36888	**FLT3**	-0.046	-0.243	-0.233	-0.157	0.133	0.358
Q15303	**HER4**	-0.040	-0.223	-0.267	0.093	0.025	0.392

Table S5. Symbol key for network modeling of altered kinases with MetaCore

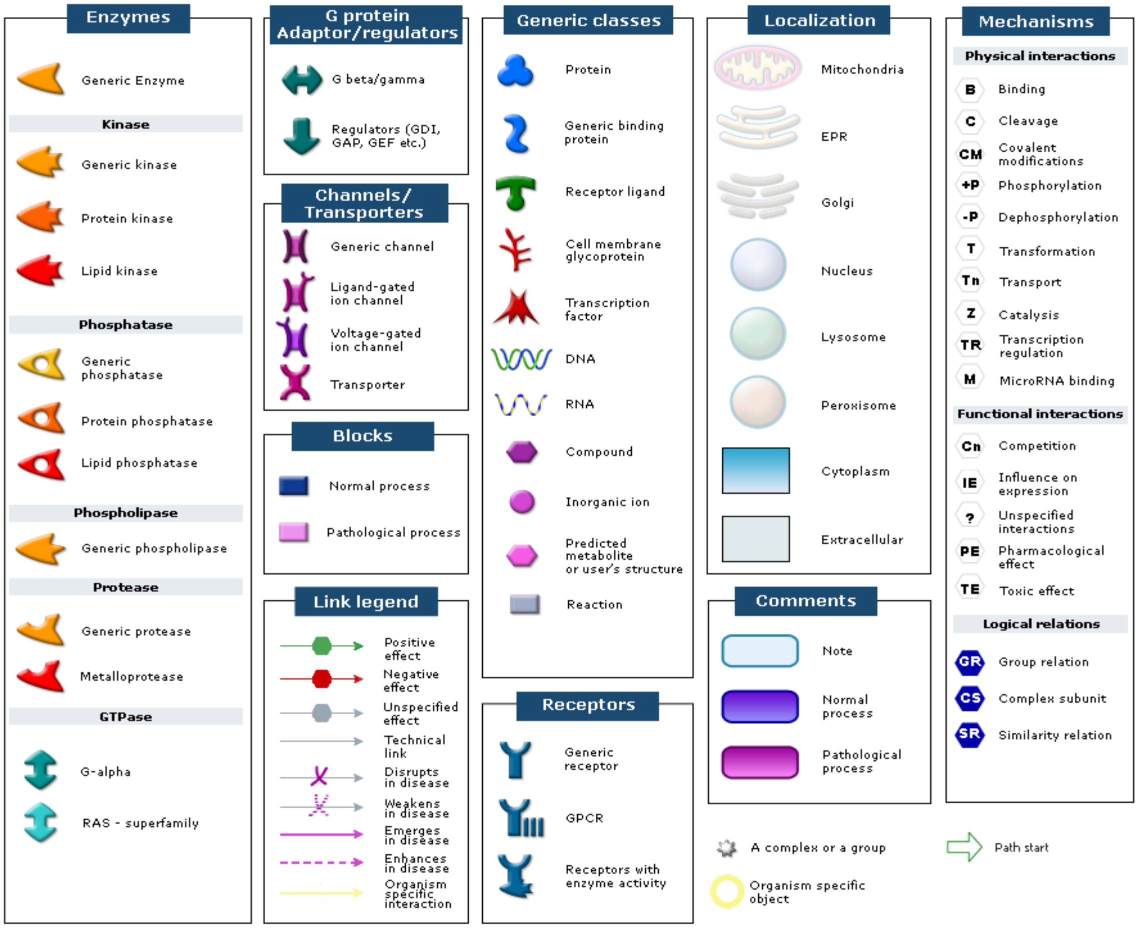

Supplement: Supplementary file 2 — Additional file 2: Supplementary Tables: Table S1. Genes status in CRC cell lines, Table S2. List of antibodies used in this study, Table S3. Primer Sequences for validation of gene targets Table S4. Mean Kinase Statistic in SW480 and RKO cell lines treated with JAK/HDACi and regorafenib and their combination compared their respective control (DMSO treatment), Table S5. Symbol key for network modeling of altered kinases with MetaCore. [file 13046_2024_3106_MOESM2_ESM.pdf]
